# P-1377. Rates of Screening of Sexually Transmitted Infections in Male Military Trainees who Present with Dysuria

**DOI:** 10.1093/ofid/ofae631.1553

**Published:** 2025-01-29

**Authors:** Catherine Sophia Malone, Angela Osuna, Erin Winkler, Ga O Jung, Heather Yun, Joseph Marcus

**Affiliations:** San Antonio Uniformed Services Health Education Consortium, San Antonio, Texas; BAMC, San Antonio, Texas; BAMC, San Antonio, Texas; 559th Medical Group, JBSA-Lackland, Texas; Brooke Army Medical Center, San Antonio, Texas; Brooke Army Medical Center, San Antonio, Texas

## Abstract

**Background:**

A high burden of sexually transmitted infections (STIs) exists among trainees entering United States Air Force Basic Military Training (BMT). While women entering training are universally screened for STIs, men, who account for the majority of trainees, are rarely tested. The rates of testing among symptomatic men who present with dysuria during BMT are unknown. Furthermore, rates of follow-up testing among male service members who present with dysuria during BMT are unknown.

**Figure:**
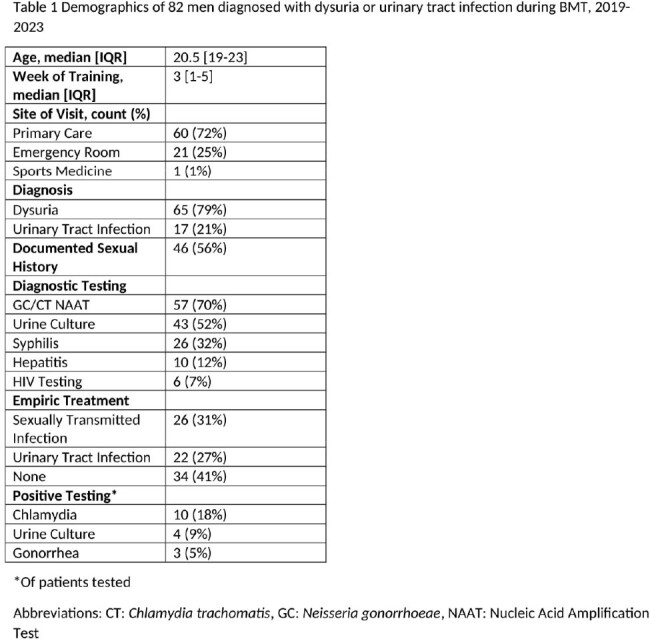

Demographics of 82 men diagnosed with dysuria or urinary tract infection during BMT, 2019-2023

**Methods:**

All male basic trainees who received a diagnostic code of dysuria or urinary tract infection between 2019-2023 were retrospectively analyzed to evaluate their diagnostic workup and follow-up over the three years after diagnosis. To assess the impact of presumptive STI therapy on subsequent follow-up, patients who received empiric treatment for STIs were compared to those who did not, with a focus on evaluating future STI testing rates and incidence. Nominal variables were compared using Fisher’s Exact Test while continuous variables were analyzed using the Mann-Whitney U test.

**Figure:**
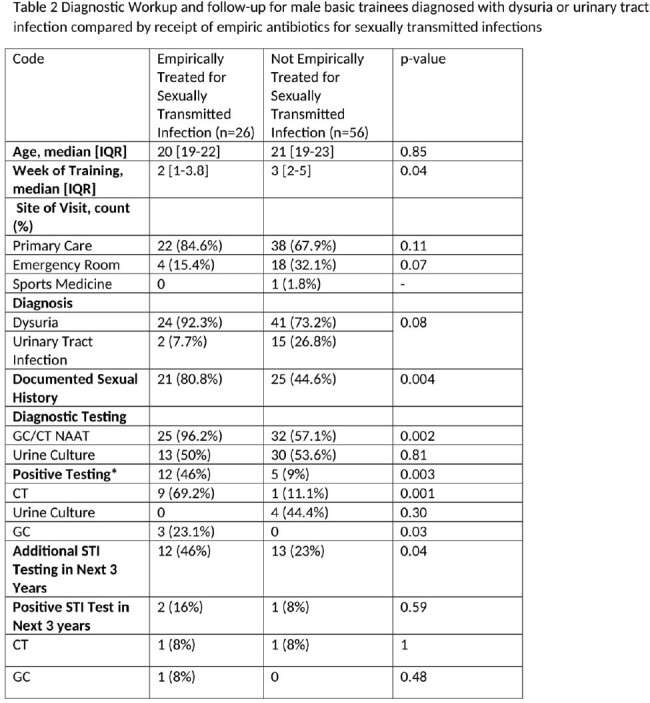

Diagnostic Workup and follow-up for male basic trainees diagnosed with dysuria or urinary tract infection compared by receipt of empiric antibiotics for sexually transmitted infections

**Results:**

Of the 102,382 men who entered BMT during the study period, 82 (0.08%) were diagnosed with dysuria or a urinary tract infection during training. Dysuria accounted for the majority of diagnoses (79%) (Table 1). 70% of symptomatic men were tested for gonorrhea and chlamydia (GC/CT). Among those tested, 23% had a positive GC/CT test. Only 25 (30%) received any additional follow-up STI testing in their next 3 years in the military, with a positivity rate of 12%. Those who were empirically treated for STIs were more likely to have a documented sexual history (80% vs. 45%, p=0.004), receive a GC/CT test (96% vs. 57%, p=0.002), and have additional STI testing in the 3 years after diagnosis (46% vs. 23%, p=0.04) (Table 2).

**Conclusion:**

There is limited data on the diagnostic workup and follow-up of men presenting with symptoms of dysuria during BMT. This study found that despite a high positivity rate for STIs in symptomatic men, sexual history documentation and STI screening were inconsistent in this population. As males account for the vast majority of basic trainees, efforts to accurately capture the burden of STIs are imperative to limit the impact on military populations.

**Disclosures:**

**All Authors**: No reported disclosures

